# The effects of liraglutide on both hypereosinophilic insulin allergy and the characteristics of anti-insulin antibodies in type 2 diabetes mellitus: a case report

**DOI:** 10.1186/s13256-016-0994-4

**Published:** 2016-07-25

**Authors:** Hiroyuki Hirai, Emi Ogata, Nobuyuki Kikuchi, Teruyuki Kohno, Noritaka Machii, Koji Hasegawa, Tsuyoshi Watanabe, Hiroaki Satoh

**Affiliations:** 1 Department of Diabetology, Endocrinology, and Metabolism, Fukushima Medical University, Fukushima, 960-1295 Japan; 2 Department of Dermatology, Fukushima Medical University, Fukushima, 960-1295 Japan

**Keywords:** Liraglutide, Insulin allergy, Human insulin-specific immunoglobulin E, Hypereosinophilia, Anti-insulin immunoglobulin G antibodies

## Abstract

**Background:**

Liraglutide is one of the glucagon-like peptide-1 analogs; there are only a few reports of liraglutide being used for the treatment of insulin allergy. Furthermore, anti-insulin immunoglobulin G antibodies are occasionally detected in patients with diabetes. Hence, we report a case in which switching to liraglutide therapy ameliorated both the symptoms of insulin allergy with hypereosinophilia and the characteristics of insulin antibodies in a patient with type 2 diabetes mellitus.

**Case presentation:**

We present the case of a 70-year-old Japanese man with type 2 diabetes who developed insulin allergy with hypereosinophilia. Anti-insulin antibodies, high glycated hemoglobin levels (approximately 12 %), and high serum insulin levels were detected. Because a change in his insulin treatment was inefficient, treatment with liraglutide to protect residual insulin secretion was started, resulting in improvements in his insulin allergy, serum glycated hemoglobin, insulin, and eosinophil levels. Scatchard plots revealed decreased binding capacity and increased affinity constant for high affinity sites of anti-insulin antibodies.

**Conclusions:**

Liraglutide might be useful for treating insulin allergy and anti-insulin antibodies in patients with type 2 diabetes.

## Background

Insulin allergy is an important adverse effect of insulin treatment in patients with diabetes. Ghazavi and Johnston recently reported that the prevalence of allergic reactions to insulin products is approximately 2 % at present [1]. Although the frequency of insulin allergy is low, the symptoms vary, ranging from local injection site reactions to complete anaphylactic reactions [2, 3]. Given that anaphylactic reactions are occasionally life-threatening, clinicians must act quickly and appropriately when allergy is suspected.

Glucagon-like peptide-1 (GLP-1) analogs have recently been introduced worldwide as a therapeutic option for the treatment of type 2 diabetes. Liraglutide is one such analog that improves blood glucose control by directly affecting both insulin and glucagon secretion in the pancreas [4]. However, there are only a few reports of liraglutide being used for the treatment of insulin allergy, and its effectiveness remains to be completely elucidated in this context [5, 6]. Furthermore, anti-insulin immunoglobulin G (IgG) antibodies [5, 7, 8] and anti-insulin receptor antibodies [5, 9] are occasionally detected in patients with diabetes who have brittle glycemic control or severe insulin resistance. The effectiveness of liraglutide remains unclear in these cases.

We report a case in which switching to liraglutide therapy ameliorated both the symptoms of insulin allergy with hypereosinophilia and the characteristics of insulin antibodies in patients with type 2 diabetes mellitus.

## Case presentation

### History leading to admission

Our patient is a 70-year-old Japanese man with diabetes. He was admitted to our hospital 4.5 years ago for further investigation and treatment of recent-onset insulin allergy. He had a 30-year history of type 2 diabetes mellitus and an approximately 6-year history of insulin injection administration, with an average glycated hemoglobin (HbA1c) level of 7.0 %. He reported a past history of myocardial infarction, hypertension, and dyslipidemia and had undergone coronary artery bypass grafting at 68 years. During that surgery, intravenous protamine sulfate was administrated, which induced anaphylaxis.

At that time, 4.5 years prior to the current presentation, he was receiving 100 mg/day aspirin, 100 mg/day clopidogrel, 100 mg/day imidapril, 20 mg/day isosorbide mononitrate, 5 mg/day nicorandil, and 10 mg/day pravastatin, and these had been stable for more than 3 years. In addition, he was being treated with 20 U/day insulin lispro and 8 U/day insulin glargine, which had also been unchanged for approximately 3 years. Finally, he had been receiving 0.9 mg/day voglibose to supplement his insulin therapy for approximately 5 years.

**Fig. 1:**
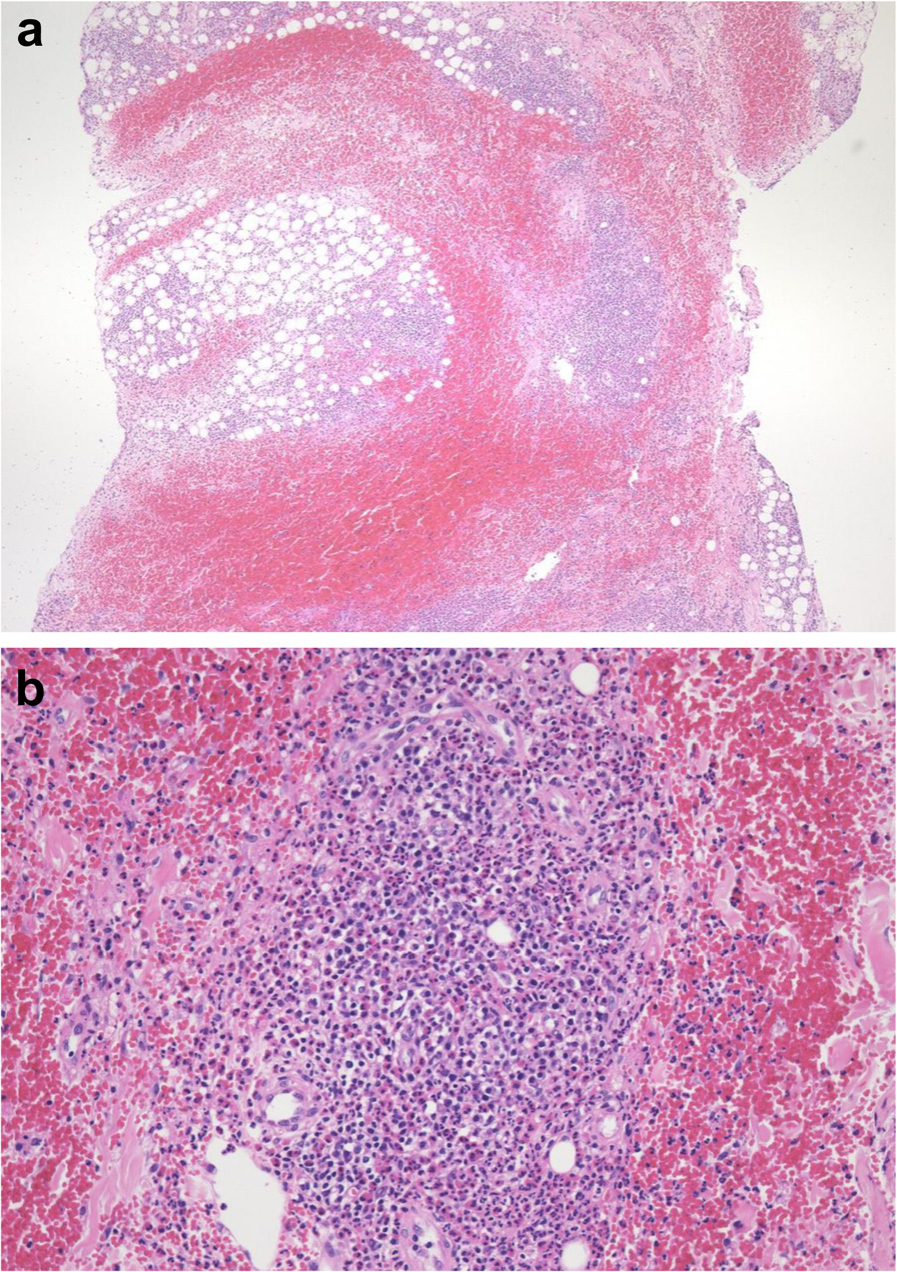
Pathological findings of insulin injection site. Specimen obtained from the injection site of a 70-year-old man with diabetes. Hematoxylin–eosin staining showing predominant infiltration of eosinophils and the simultaneous infiltration of lymphocytic cells in the subcutaneous tissue. **a** Low-power micrograph 40×. **b** High-power micrograph 200×

Approximately 5 months before his current presentation, he reported that he had started to develop wheals with redness and itching at the injection site, which occurred immediately after injecting insulin. Of note, these symptoms only occurred following the use of insulin lispro and not after insulin glargine. Over the subsequent months, part of the surface of the wheals had hardened and further investigation was performed. Two months before his current admission, blood samples were taken that revealed high serum levels of human insulin-specific immunoglobulin E (IgE; 4.21 U/mL; normal range <0.35 U/mL), hypereosinophilia (total white blood cell count, 6000/μL; total eosinophil granulocyte count, 780; percentage, 13 %), and a high level of total IgE (403 IU/mL; normal range <173 IU/mL). A pathology sample was taken 2 months before his admission, which revealed eosinophilic infiltration in the hardened part of the injection site (Fig. 1). Therefore, just before admission, he was diagnosed with insulin allergy and was switched from insulin lispro plus glargine to insulin aspart plus glargine.

### Admission for further investigation

He was admitted to our hospital for treatment of his poor glycemic control and to evaluate his residual ability of insulin secretion. On admission, his physical examination was largely unremarkable (height, 157.1 cm; body weight, 53.0 kg; body mass index, 21.5 kg/m^2^; body temperature, 36.5 °C; blood pressure, 104/62 mmHg; and pulse, 75 beats/minute, regular), although there was induration with slight redness of his abdomen. His laboratory data at that time are shown in Table 1. Of note, his fasting plasma glucose level was 346 mg/dL, and his HbA1c level was 12.3 %. In addition, we detected high serum insulin, anti-insulin IgG antibody, and anti-insulin receptor antibody levels. Although there were no further symptoms of local allergy (wheals, redness, and itching) after injecting with insulin aspart, his postprandial hyperglycemia continued at approximately 350 mg/dL. Therefore, he was switched to insulin glulisine (24 U/day) plus glargine (22 U/day) after admission. On day 17 of admission, his 24-hour blood glucose profile was 88, 241, 241, 295, 297, and 292 mg/dL at 7:30 a.m., 10:00 a.m., 11:30 a.m., 2:00 p.m., 5:30 p.m., and 8:00 p.m., respectively, with an energy intake of 1600 kcal. On days 18 and 19, his urinary excretion of C-peptide was 42.8 to 51.5 μg/day (normal range 40 to 100 μg/day). On day 19, a glucagon stimulation test was performed, and his serum C-peptide response ranged from 3.2 to 3.6 ng/mL (Table 2). On day 22, although his high glucose levels persisted, he was discharged because we had confirmed his ability to secrete insulin. Although his hypereosinophilia was not resolved before his discharge, he had no further allergic symptoms (wheals, redness, and itchiness) at the injection site for glulisine, and the area of induration had almost disappeared.

**Table 1:** Laboratory data on admission

Parameter	Result (normal range)	Unit	Parameter	Result (normal range)	Unit
WBC	5400 (2800–8800)	/μL	γGTP	32 (10–47)	IU/L
Neutrophil	60 (32–79)	%	Ch-E	378 (214–466)	IU/L
Eosinophil	15 (0–6)	%	T-Bil	0.9 (0.2–1.2)	mg/dL
Lymphocyte	16 (18–59)	%	Na	138 (138–146)	mEq/L
Monocyte	7(0–8)	%	K	4.9 (3.6–4.9)	mEq/L
Basophil	2(0–2)	%	Cl	99 (99–109)	mEq/L
RBC	406×10^4^ (366–478)	/μL	BUN	19 (8–22)	mg/dL
Hb	13.0 (11.6–14)	g/dL	Cre	1.0 (0.4–0.7)	mg/dL
Hct	38.0 (34.1–41.7)	%	UA	4.7 (2.3–7)	mg/dL
Plt	15.2×10^4^ (14.7–34.1)	/μL	CK	209 (62–287)	IU/L
			AMY	46 (42–132)	IU/L
TP	7.2 (6.7–8.3)	g/dL	CRP	0.62 (0–0.3)	mg/dL
Alb	3.6 (3.9–4.9)	g/dL	TC	149 (150–219)	mg/dL
AST	33 (13–33)	IU/L	TG	70 (30–150)	mg/dL
ALT	28 (6–27)	IU/L	HDL-C	50 (49–74)	mg/dL
FPG	346 (70–109)	mg/dL	ANA	Negative (<40×)	
F-INS	9774 (5–10)	μIU/mL	CH50	<12 (25-48)	CH50/mL
F-CPR	4.9 (0.6–2.1)	ng/mL	IgG	1697 (870–1700)	mg/dL
HbA1c	12.3 (4.7–6.2)	%	Anti-SS-A antibody	Negative	
Glycoalbumin	48.3 (12.4–16.3)	%	Anti-SS-B antibody	Negative	
GAD antibody	0.3 (0–5.0)	U/mL	Anti-ds-DNA antibody	Negative	IU/mL
Human IgE	5.57 (0–0.34)	U/mL	RF	<3 (<15)	IU/mL
IgG-anti-insulin antibodies	14.5 (<0.3)	%			
Anti-insulin receptor antibodies	Positive		Urine test		
ACTH	11.6 (7.2–63.3)	pg/mL	U-Glucose	(4+)	
Cortisol	17.9 (6.2–19.4)	μg/dL	U-Protein	Negative	
TSH	1.747 (0.5–5.0)	μIU/mL	U-Blood	Negative	
FT4	2.11 (0.9–1.7)	ng/dL	U-Ketone	Negative	

*ACTH* adrenocorticotropic hormone, *Alb* albumin, *ALT* alanine aminotransferase, *AMY* amylase, *ANA* anti-nuclear antibody, *Anti-ds-DNA* anti-double-stranded DNA, *anti-SS-A* anti-Sjögren’s syndrome A, *anti-SS-B* anti-Sjögren’s syndrome B, *AST* aspartate aminotransferase, *BUN* blood urea nitrogen, *Ch-E* cholinesterase, *CK* creatine, *Cl* chloride, *Cre* creatinine, *CRP* C-reactive protein, *F-CPR* fasting C-peptide, *F-INS* fasting insulin, *FPG* fasting plasma glucose, *FT4* free thyroxine, *GAD* glutamic acid decarboxylase, *γ-GTP* γ-glutamyl transpeptidase, *Hb* hemoglobin, *HbA1c* glycated hemoglobin, *Hct* hematocrit, *HDL-C* high density lipoprotein cholesterol, *IgE* immunoglobulin E, *IgG* immunoglobulin G, *K* potassium, *Na* sodium, *Plt* platelets, *RBC* red blood cells, *RF* rheumatoid factor, *T-Bil* total bilirubin, *TC* total cholesterol, *TG* triglyceride, *TP* total protein, *TSH* thyroid-stimulating hormone, *UA* uric acid, *WBC* white blood cells

**Table 2:** An evaluation of the patient’s insulin secretion ability

(a) Glucagon stimulation test		
Time (minutes)	0	6
PG (pg/mL)	112	122
CP (ng/mL)	3.2	3.6
(b) Urinary C-peptide excretion		
Day 18 (after admission)	Day 19	Normal range
42.8 μg/day	51.5 μg/day	(40–100)

*CP* C-peptide, *PG* plasma glucose

### Progress since discharge and the introduction of liraglutide

**Fig. 2:**
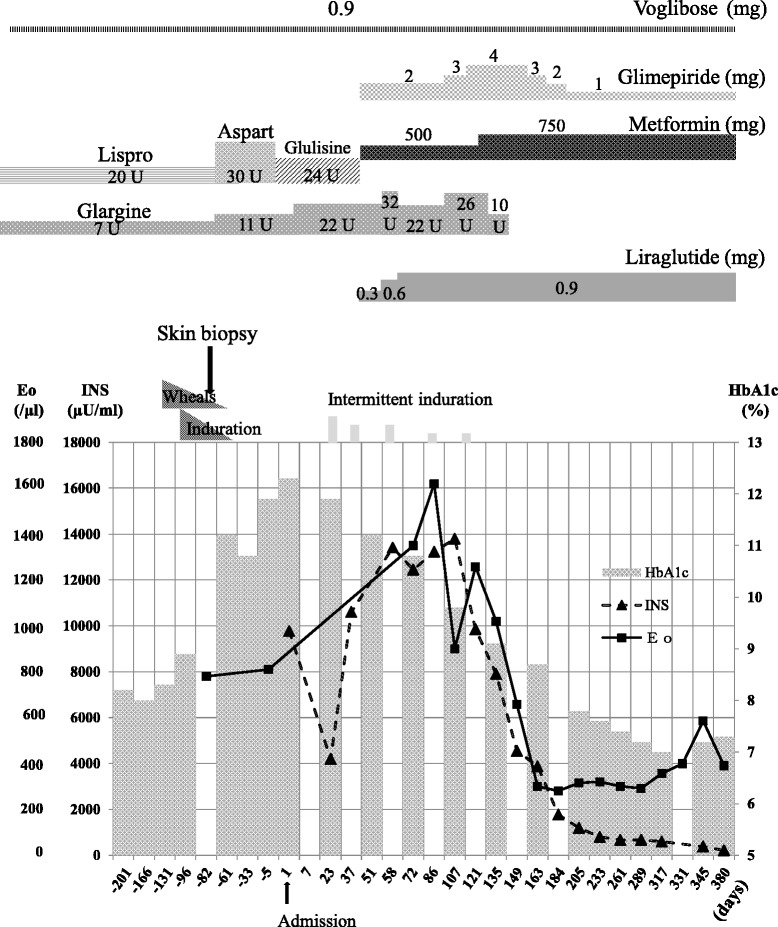
Clinical course. A diagrammatic representation of the treatment and clinical course of a 70-year-old man with insulin allergy. He presented 5 months before his admission with wheals, redness, and itching immediately after insulin lispro injection that progressed to an area of induration. A skin biopsy and blood tests confirmed an allergy, and after trialing different insulin regimens, we eventually introduced 0.3 mg liraglutide, discontinued the short-acting insulin, and added glimepiride and metformin. However, induration intermittently occurred after insulin glargine injections, so we eventually cross-tapered him off glargine and onto a liraglutide dose of 0.9 mg. Thereafter, his glycated hemoglobin, insulin, and eosinophil levels gradually improved. *Eo* eosinophil count, *HbA1c* glycated hemoglobin, *INS* insulin dose

Figure 2 summarizes his clinical course before and after the introduction of liraglutide. After discharge, although glulisine and glargine were continued, his glycemic control worsened, with fasting and postprandial hyperglycemia levels of approximately 200 and 300 mg/dL, respectively, and high serum insulin levels persisted (Fig. 2). Therefore, 2 months after his admission, we added 0.3 mg liraglutide, discontinued the insulin glulisine, and added 2 mg/day glimepiride and 500 mg/day metformin to decrease his total insulin requirements. However, he also started to develop intermittent induration at the site of insulin glargine injection, and although there was no evidence of wheals or redness, his hypereosinophilia persisted. Based on these findings, we reduced the dose of glargine (32→22→26→10 U/day) and increased the dose of liraglutide (0.3→0.6→0.9 mg/day; Fig. 2). This was followed by improvements in his HbA1c, serum insulin, and eosinophil levels; therefore, we decided to completely discontinue the insulin glargine after 3 months of the liraglutide treatment. On his new regimen of 0.9 mg/day liraglutide in combination with oral antidiabetic agents, his HbA1c level was maintained at approximately 7.0 %. The induration had almost completely disappeared 1 month after the insulin injections were discontinued. In addition, his body weight decreased by 1.7 kg after 2 months of liraglutide treatment. Table 3 indicates his diabetes-related medical history.

**Table 3:** Timeline

Time	Diabetes-related medical history
1980s	Onset of type 2 diabetes mellitus
2009	Coronary artery bypass grafting was conducted, anaphylaxis induced by intravenous protamine sulfate administration
May 2011	Onset of allergy
July 2011	A skin biopsy and blood tests confirmed an allergy
24 October 2011	Admission to our hospital
14 December 2011	Liraglutide was additionally started
21 March 2012	Insulin discontinued
April 2012	The symptoms of insulin allergy almost disappeared

### Supplemental investigation by Scatchard analysis

**Fig. 3:**
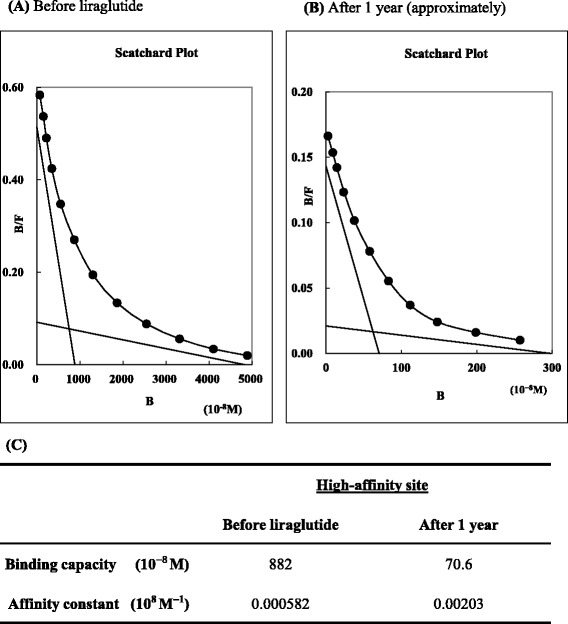
Scatchard plot analysis showing changes in the characteristics of anti-insulin immunoglobulin G antibodies before and after treatment with liraglutide. **a** The Scatchard plot analysis before the treatment of liraglutide. The binding capacity (10^−8^ M) of 882 (R_1_) and affinity constant (10^8^ M^−1^) of 0.000582 (K_1_) for high affinity sites and the binding capacity (10^−8^ M) of 4830 (R_2_) and affinity constant (10^8^ M^−1^) of 0.0000190 (K_2_) for low affinity sites are shown. **b** The Scatchard plot analysis approximately 1 year after starting liraglutide. The binding capacity (10^−8^ M) of 70.6 (R_1_) and affinity constant (10^8^ M^−1^) of 0.00203 (K_1_) for high affinity sites and the binding capacity (10^−8^ M) of 298 (R_2_) and affinity constant (10^8^ M^−1^) of 0.0000704 (K_2_) for low affinity sites are shown. **c** It is notable, particularly for high affinity sites, that the binding capacity (10^−8^ M) has changed from 882 to 70.6 (R_1_), and that the affinity constant (10^8^ M^−1^) has changed from 0.000582 to 0.00203 (K_1_). Therefore, liraglutide appeared to induce a decrease in the binding capacity and an increase in the affinity constant for high affinity sites of anti-insulin immunoglobulin G antibodies

We also elucidated the changes in the characteristics of anti-insulin IgG antibodies before and after treatment with liraglutide. Scatchard analysis of the anti-insulin IgG antibodies in our case indicated that there was a high binding capacity and a low affinity constant for the high affinity sites before liraglutide therapy (Fig. 3). After 1 year of treatment with liraglutide, the Scatchard plot indicated that the binding capacity decreased and the affinity constant for high affinity sites increased (Fig. 3). Of interest, the anti-insulin receptor antibodies had completely disappeared within 1 year after the injection of liraglutide.

## Discussion

There are two important aspects to this case. First, we provide a rare description of insulin allergy associated with hypereosinophilia, anti-insulin IgG antibodies, and anti-insulin receptor antibodies in a patient with type 2 diabetes. Second, the marked efficacy of liraglutide suggests that it is useful in all patients with type 2 diabetes.

In our case, we believed that our patient’s insulin allergy was predominantly a type 1 immediate reaction based on the fact that insulin lispro induced wheals, itching, and redness immediately after injection. Insulin allergy is typically classified into the following: type 1, which is local and immediate; type 3, which is related to autoimmune disease; and type 4, which is a delayed-type reaction. Symptoms typically manifest within 15 minutes after insulin injection in type 1 reactions, approximately 6 hours after type 3 reactions, and 8 to 24 hours after type 4 reactions [2]. In our case, we confirmed that insulin lispro induced immediate symptoms but could not detect whether insulin aspart, glulisine, or glargine induced those reactions.

We did not perform either a skin prick test or an intradermal reaction test because of his past history of anaphylaxis. Out of concern for his safety, we substituted this with a non-invasive drug-induced lymphocyte stimulation test, which is normally considered best suited for delayed (type 4) reactions. Although this test was only positive for insulin glulisine, false-negatives have been detected in some cases, and we considered that insulin glargine was also associated with delayed-type allergy. This was because insulin glargine induced occasional induration and because its discontinuation resulted in improvement of both his induration and hypereosinophilia.

Based on the combination of these results, we concluded that our patient probably had a delayed-type allergic reaction that was complicated with a predominant immediate-type allergic reaction. Unfortunately, we did not obtain the list of additives in either the lispro or glargine, which could have been important given his history of protamine anaphylaxis. Further investigation of the cause of the allergic reactions could not be performed (for example, to other insulin preparations, including human neutral protamine Hagedorn, or the additives in the insulin types).

Previous reports of type 1 and type 4 allergic reactions to insulin indicate that hypereosinophilia does not necessarily occur. Therefore, the increase of eosinophilia in both local pathological findings and systemic peripheral blood is characteristic of this case. In general, although numerous eosinophils tend to be detected in local inflammation, such as allergy and parasitic infection, the complete role of eosinophils has not been elucidated [10]. For example, although hypereosinophilia has been observed in invasive parasitic infection, it has recently been reported to be partially defensive *in vivo* during such an infection [10]. In clinical cases of hypereosinophilia, clinicians must quickly and accurately diagnose the cause [11]. Potential causes in the differential diagnosis of this case included adrenal crisis, parasitic infection, and blood disease, but there was no evidence in support of these diagnoses. In addition, because hypereosinophilia simultaneously occurred with the observed clinical course of insulin allergy, we considered insulin allergy the more likely cause. This was consistent with the report by Nagai *et al*., who reported a case of immediate-type allergy against human insulin associated with marked eosinophilia in a patient with type 2 diabetes [12]. In that case, there was also the possibility of hypereosinophilia-induced renal dysfunction. Although there was no evidence of organ damage in this case, clinicians should remain vigilant for hypereosinophilic syndrome [11, 13] and be prepared to initiate appropriate therapy. Currently, treatment options for insulin allergy include switching to another insulin device [2], steroid therapy [2], continuous subcutaneous insulin infusion therapy [14], hyposensitization therapy [15], pancreas transplantation [16], and anti-IgE therapy [17]. However, reports on the treatment of insulin allergy with hypereosinophilia are limited, and new options are needed. Therefore, we consider that liraglutide might be a breakthrough agent in the treatment of insulin allergy with hypereosinophilia in patients with type 2 diabetes. Indeed, a Medline search indicated that at the time of writing, we are the first to report the beneficial effects of liraglutide in this setting.

Treatment with liraglutide requires the residual ability to secrete insulin, but we do not know the appropriate range of insulin secretion that is necessary. Previously, Kozawa *et al*. reported that the cut-off value for predicting the efficacy of liraglutide was 1.1 for the C-peptide index, which was defined as (fasting C-peptide/glucose)×100, and 1.5 ng/mL for fasting C-peptide [18]. This index was also shown to be a predictive marker for the beta-cell area of the human pancreas [18, 19]. Using a glucagon stimulation test, Usui *et al*. reported that a ΔC-peptide value of 1.34 ng/mL was a useful cut-off point for switching from insulin to liraglutide without developing hyperglycemia [20]. In this case, although the fasting C-peptide level was high, the ΔC-peptide in the glucagon stimulation test had a low value. Therefore, liraglutide alone could not inhibit postprandial hyperglycemia, and the antidiabetic drugs glimepiride, metformin, and voglibose were combined to achieve complete control. Moreover, the additional benefits of liraglutide include the prevention of body weight gain due to its appetite-suppressing and gastrointestinal peristaltic movement-suppressing effects. Liraglutide and other GLP-1 analogs have been reported to decrease body weight [21, 22]. In our case, our patient’s body weight decreased by only 1.7 kg, but we could not administer liraglutide at a dose of more than 0.9 mg/day. Worldwide, liraglutide is administered at a maximum dose of 1.8 mg/day; however, Japan has approved that liraglutide is administered at a maximum dose of only 0.9 mg/day.

In our case, it is possible that both the insulin allergy and the presence of anti-insulin IgG antibodies themselves aggravated our patient’s glycemic control. Therefore, we tested his human leukocyte antigen-antigen D related (HLA-DR) type based on the report by Uchigata *et al*. that insulin autoimmune syndromes are strongly associated with HLA-DR4 [23]. We showed that his HLA-DR types were DR-9 and DR-15 and not DR-4; thus, we did not consider HLA-DR to be a potential causative factor for producing anti-insulin IgG antibodies. Instead, we concluded that the anti-insulin IgG antibodies resulted from the exogenous insulin injection; thus, we used liraglutide without insulin to decrease the amount of anti-insulin IgG antibodies and to avoid further production.

Moreover, Scatchard analysis of the anti-insulin IgG antibodies in our patient indicated that there was a high binding capacity and a low affinity constant for high affinity sites. At high affinity sites, Eguchi reported two important patterns of Scatchard analysis of anti-insulin IgG antibodies: (1) high (11.5 to 53.2) binding capacity (10^−8^ M) and low (0.04 to 0.21) affinity constant (10^8^ M^−1^) in insulin autoimmune syndrome; and (2) low (0.12 to 1.1) binding capacity (10^−8^ M) and high (1.45 to 7.11) affinity constant (10^8^ M^−1^) in patients with diabetes requiring insulin therapy [24]. Therefore, the binding capacity and low affinity constant for high affinity sites are clinically important. Our patient had extremely high binding capacity and a low affinity constant compared with previous reports. In particular, his extremely high binding capacity was consistent with his high serum insulin levels.

After insulin therapy was discontinued and liraglutide was started, the characteristics of his anti-insulin IgG antibodies changed. Specifically, there was a notable decrease in the binding capacity and an increase in the affinity constant for high affinity sites (Fig. 3c). Hara *et al*. have reported that antibody-mediated insulin resistance was treated by ceasing insulin administration [25], whereas Tamura *et al*. reported that liraglutide ameliorated the anti-insulin IgG antibodies in a woman with type 2 diabetes receiving hemodialysis [26]. In our case, it remains unclear how liraglutide might have affected our patient’s anti-insulin IgG antibodies; however, a recent report has suggested that GLP-1 receptor agonists interact with the immune system. In fact, Hadjiyanni *et al*. reported that GLP-1 receptor signaling selectively regulated murine lymphocyte proliferation and maintained peripheral regulatory T cells *in vivo* [27]. In humans, the effects of GLP-1 receptor agonists have been investigated in patients with psoriasis [28], but the detailed mechanism of action is unclear. Further reports are needed to improve our understanding of these agents.

Anti-insulin IgG antibodies have often been reported to induce hypoglycemia [8, 21, 23, 24]. Unfortunately, we could not measure serum free insulin levels directly and do not know whether the anti-insulin receptor antibodies really affected glucose metabolism. Although Kim *et al*. reported that anti-insulin receptor antibodies often coexist with anti-insulin IgG antibodies in Korean patients with insulin autoimmune syndrome [29], the clinical significance of this finding has been not determined. Acquired anti-insulin receptor antibodies induce severe insulin resistance, called “type B insulin resistance,” which was first reported in 1976 [30]. However, we found no other symptoms or complications of type B insulin resistance, such as acanthosis nigricans or autoimmune disease (Table 1). In addition, the total insulin dose was smaller than that reported in previous studies on type B insulin resistance [9, 30]. Therefore, we considered the possibility that the presence of anti-insulin receptor antibodies is a false positive in this case. It is also possible that the method of measurement led to a false positive result for anti-insulin receptor antibodies because we used the insulin binding inhibition method [31]. Perhaps the high insulin levels and high anti-insulin IgG antibodies levels had some unknown influence on the measured values.

## Conclusions

In conclusion, our case suggests that liraglutide is a useful treatment for insulin allergy associated with hypereosinophilia and anti-insulin IgG antibodies in patients with type 2 diabetes. However, similar case reports are limited, and further reports are clearly needed.

## Abbreviations

GLP-1, glucagon-like peptide-1; HbA1c, glycated hemoglobin; HLA-DR, human leukocyte antigen-antigen D related; IgE, immunoglobulin E; IgG, immunoglobulin G
